# GENDER AND AGE DIFFERENCES IN ACCEPTANCE OF ADVANCED DRIVER ASSISTANCE SYSTEMS: INSIGHTS FROM OLDER AUSTRALIANS

**DOI:** 10.1093/geroni/igad104.1352

**Published:** 2023-12-21

**Authors:** Abigail Hansen, Kim Kiely, Tuki Attuquayefio, Diane Hosking, Michael Regan, Ranmalee Eramudugolla, Lesley Ross, Kaarin Anstey

**Affiliations:** University of New South Wales, Sydney, New South Wales, Australia; University of New South Wales, Sydney, New South Wales, Australia; UNSW, Sydney, New South Wales, Australia; National Seniors Australia, Canberra, Australian Capital Territory, Australia; University of NSW Sydney, Sydney, New South Wales, Australia; University of New South Wales, Sydney, New South Wales, Australia; Clemson University, Clemson, South Carolina, United States; University of New South Wales, Sydney, New South Wales, Australia

## Abstract

Advanced Driver-Assistance Systems (ADAS) are in-vehicle technologies that promise to improve driver safety and may help support older drivers to drive safer for longer, however there is little research examining acceptance and use of ADAS among older adults. This study investigated age and gender differences in attitudes to ADAS and use of ADAS. We conducted an online survey of 1330 drivers aged 65 years or older (M=72, SD=6.9, 23% women) in partnership with National Seniors Australia. ADAS use was self-reported, classifying respondents in to users (96%) and non-users (4%). ADAS acceptance was measured by the Partial Automation Acceptance Scale. Linear regression estimated gender and age differences in acceptance. Logistic regression estimated differences in ADAS use by gender, age and acceptance. Older age was associated with higher levels of trust in ADAS (β = 0.032, p = 0.04) and lower perceived ease of use (β = -0.02, p = 0.02). Women were less likely to report ADAS use (β = -0.99, p = 0.008) but had higher levels of trust (β = 0.86, p = 0.001) than men. After adjusting for age and gender, positive attitudes towards ADAS (β = 0.12, p = 0.003) and lower perceived risk (β = 0.22, p = 0.046) were associated with higher levels of ADAS use. Consideration of the gender and age differences may inform future vehicle design, and older drivers may benefit from education on the risks and benefits of ADAS to aid in the acceptance and consequent use of ADAS.

